# Increased expression of protease-activated receptors 2 indicates poor prognosis in HBV related hepatocellular carcinoma

**DOI:** 10.1186/s13027-019-0256-3

**Published:** 2019-11-21

**Authors:** Peng Chen, Na Yang, Li Xu, Fangfang Zhao, Min Zhang

**Affiliations:** 1 Gastroenterology Department, Shandong Zaozhuang Mining Group Central Hospital, Zaozhuang, Shandong China; 2 Blood transfusion department of Zaozhuang Maternal and Child Health Hospital, Zaozhuang, Shandong China; 3 Purchasing department of Shandong Zaozhuang Mining Group Central Hospital, Zaozhuang, Shandong China; 4 Liver Diseases Department, Shandong Zaozhuang Mining Group Central Hospital, Zaozhuang, Shandong China; 5 Department of Gastroenterology, Qingdao No.6 People’s Hospital, No. 9 Fushun Road, Sifang District, Qingdao, Shandong China

**Keywords:** Protease-activated receptors 2, Prognosis, Hepatocellular carcinoma, Biomarker

## Abstract

**Objective:**

To investigate the potential role of protease-activated receptor 2 (PAR2) in the prognosis of hepatocellular carcinoma (HCC).

**Methods:**

A total of 202 HCC patients who underwent liver resections were included. Tissue microarray was established with specimens of both HCC and paired adjacent liver tissues. PAR2 expression was detected by immunohistochemistry (IHC) assays.. A semi-quantification method was used to define the expression level of PAR2. The correlations between PAR2 expression and clinical features of patients with HCC was explored. The association of different PAR2 expressions with both overall survival and disease-free survival was analyzed.

**Results:**

Results showed that the expression of PAR2 in HCC tissues was higher than that in paired para-cancerous liver tissues (4.12 ± 3.55 vs. 2.71 ± 2.56, *P* < 0.001). Higher expression of PAR2 was associated with poor differentiation (P < 0.001) and advanced tumor-node-metastasis stage (*P* = 0.015). Kaplan-Meier survival analysis indicated that HCC patients with high PAR2 expression had decreased overall survival (*P* = 0.033) and disease-free survival (*P* = 0.043) compared to patients with lower PAR2 expression. Multivariate analysis indicated that PAR2 expression (*P* = 0.032) was a significant independent prognostic factor for both overall survival and disease-free survival (P = 0.032; P = 0.032, respectively).

**Conclusion:**

Our data revealed that PAR2 expression was increased in HCC. High PAR2 expression was correlated with both decreased overall survival and disease-free survival in patients with HCC. High PAR2 expression indicated a poor prognosis.

## Introduction

Hepatocellular carcinoma (HCC) is one of the most common malignant tumors in China, as well as the sixth malignant tumor with high morbidity in the world [1–3]. Nearly 1 million new cases of HCC are diagnosed each year [4, 5]. Previous evidence revealed that chronic infection with hepatitis viruses represents major risk factor for HCC development and progression [6–8]., particularly in developing regions, such as East Asia and Africa [9–14]. Although liver resections may improve the clinical outcomes of HCC patients, the prognosis is still poor due to the high recurrence rate [15–17]. Thus, the identification of novel biomarkers to screen HCC patients with poorer clinical outcome is critical for a better clinical management.

Protease-activated receptors (PARs) belong to 7-domain transmembrane G-protein receptor families, including four subtypes, PAR1, PAR2, PAR3, and PAR4 [18, 19]. PARs are characterized by a unique activation mechanism involving protein degradation of the lanthanide ligands. Several studies indicated that PARs were associated with vascular regulation, inflammatory response, tissue fibrosis and carcinogenesis [20–22], which made PARs the potential targets for novel therapies development. It has been confirmed that PAR2 was involved in development of HCC and pancreas cancer [20, 23]. PAR2 is the only member of PAR family to be considered a tumor suppressor factor in skin tumor [22]. Whether PAR2 has a role in the prognosis of HCC remained to be explored.

Hence, we designed this study to compare survival of HBV related HCC patients with different PAR2 expression and analyze the correlation between PAR2 expression in HCC and patients’ prognosis.

### Subjects and methods

### 2.1 Subjects

202 HCC patients were included in our study. All of them received liver resections from June 2013 to July 2016. All patients were sero-positive for hepatitis B surface antigen (HBsAg) [24, 25]. All patients received first-line anti-HBV drugs (Entecavir or Tenofovir) during follow-up [26]. None of the patients received any chemotherapy or radiotherapy. The follow-up period was defined as the time interval from liver resection to the date of death or the last follow-up. The study was approved by the medical ethics committee of Qingdao No.6 People’s Hospital. Since all specimens were collected anonymously, the Medical Ethics Committee exempted patients from the need for informed consent.

### 2.2 Tissue microarray construction and immunohistochemistry

Tissue microarray (TMA) was constructed as below. In brief, each tissue core (diameter: 0.6 mm) was perforated and re-embedded from the labeled area by using a tissue array. The specimens were fixed with 4% paraformaldehyde and blocked using the biotin blocking Kit (Dark, Germany). After blocking, the tissues were incubated with PAR2 antibodies (180,953, 11,000 dilution, Abcam, England) in a humid chamber at 4 °C overnight. The tissues were washed with PBS three times and then incubated with biotinylated goat anti-rabbit antibodies for one-hour at 37 °C. Finally, the slices were stained with hematoxylin and observed under a microscope. Semi-quantitative method was used to define PAR2 protein expression levels according to the following criteria: “0” (negative staining), “1” (weak staining), “2” (moderate staining) and “3” (strong staining). The final score was calculated as the percentage of cells with staining multiplied by the intensity score. The median IHC score was used as a cut-off value for determining high and low PAR2 expression. In our study, microvascular invasion was defined as the presence of tumor cells within the vascular space lined by endothelium observed by microscopy [6, 15, 27].

### 2.3 Statistical analysis

Statistical analysis was performed using SPSS software (version 13; SPSS Inc., Chicago, IL, USA). Student’s t test or Chi square test was used to examine the correlation between PAR2 expression and clinical and pathological variables. The Kaplan-Meier method (logarithmic rank test) was used to construct the survival curve. Multivariate Cox proportional hazards regression model was used to assess the independent predictive factors. *P* value less than 0.05 was defined as statistically significant.

## Results

### 3.1 Expression of PAR2 in the HCC tissues

**Fig. 1:**
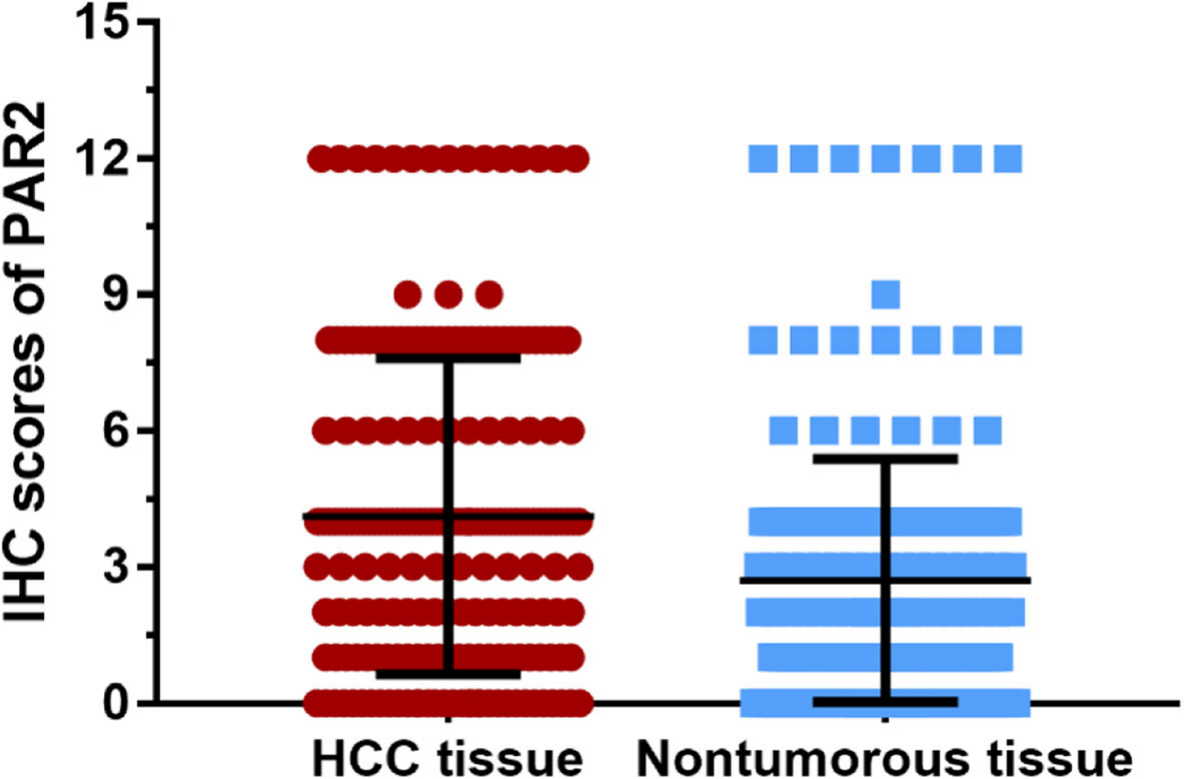
Expression of PAR2 in HCC tissues**.** The results showed that the expression level of PAR2 in HCC tissues was 4.12 ± 3.55, significantly higher than that of matched paracancerous liver tissues with 2.71 ± 2.56 (*P* < 0.001)

The expression level of PAR2 in 202 pairs of HCC tissues and matched paracancerous liver tissues were detected. The median PAR2 expression in HCC tissues was 2.0. The expression level of PAR2 in HCC tissues was 4.12 ± 3.55, significantly higher than that of matched paracancerous liver tissues (2.71 ± 2.56, *P* < 0.001, Fig. 1).

### 3.2 Association of PAR2 with HCC clinical pathologic features

We used the median PAR2 expression level to stratify HCC patients into PAR2 low expression group (< 2.0) and PAR2 high expression group (≥2.0). Demographic data and clinical features of the patients in the two groups were compared. We found that patients with high expression of PAR2 had significantly higher proportion of poor differentiation than those with low expression of PAR2 (*P* < 0.001). In addition, high expression of PAR2 was associated with advanced tumor-node-metastasis (TNM) stage (*P* = 0.015), as shown in Table 1.

**Table 1:** Clinical variables in patients with high and low expression of PAR2

Variable	PAR2 expression		*P* value
	Low expression	High expression	
Sample size	63	139	
Age, years			0.074
> 50	25 (39.7%)	74 (53.2%)	
≤50	38 (60.3%)	65 (46.8%)	
Gender			0.187
Male	57 (90.5%)	116 (83.5%)	
Female	6 (9.5%)	23 (16.5%)	
AFP, ng/mL			0.067
< 20	18 (28.6%)	24 (17.3%)	
≥ 20	45 (71.4%)	115 (82.7%)	
Cirrhosis			0.889
Yes	53 (84.1%)	118 (84.9%)	
No	10 (15.9%)	21 (15.1%)	
Tumor size, cm			0.154
< 5	23 (36.5%)	37 (26.6%)	
≥ 5	40 (63.5%)	102 (73.4%)	
Differentiation			< 0.001
Well-moderate	22 (34.9%)	12 (8.6%)	
Poor-undifferentiated	41 (65.1%)	127 (91.4%)	
TNM stage			0.015
I–II	42 (66.7%)	67 (48.2%)	
III–IV	21 (33.3%)	72 (51.8%)	
Vascular invasion			0.290
Yes	8 (12.7%)	26 (18.7%)	
No	55 (87.3%)	113 (81.3%)	

Abbreviations: *PAR2* proteinase activated receptor 2, *AFP* alpha-fetoprotein

### 3.3 Association of PAR2 expression with clinical outcomes in patients with HCC

**Fig. 2:**
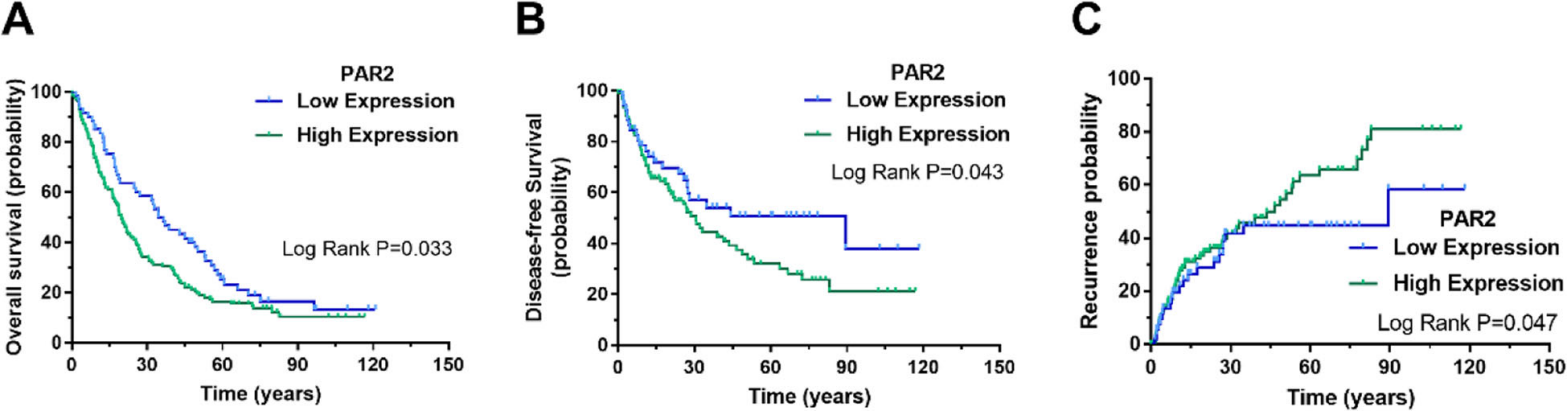
The prognostic predictive value of PAR2 in HCC**.** Kaplan-Meier survival analysis showed that HCC patients with high expression of PAR2 had a worse prognosis in overall survival than patients with low expression of PAR2 (*P* = 0.033, **a**). For disease-free survival, patients with low expression of PAR2 had a better prognosis in disease-free survival compared with patients with high expression of PAR2 (*P* = 0.043, **b**). For HCC recurrence, significantly higher proportion of recurrence in patients with high expression level of PAR2 compared with low expression group (*P* = 0.047, **c**)

In order to explore the potential impact of PAR2 expression on the survival of HCC patients, we performed a Kaplan-Meier survival analysis. The results showed that HCC patients with high expression of PAR2 had decreased overall survival (OS) compared to patients with low expression of PAR2 (*P* = 0.033). We also compared the difference in the disease-free survival (DFS) between the PAR2 high-expression group and the low-expression group. The results showed that patients with high expression of PAR2 had decreased disease-free survival compared with patients with low expression of PAR2 (*P* = 0.043). A similar trend was found in HCC recurrence, with significantly higher proportion of recurrence in patients with high expression level of PAR2 compared with low expression group (*P* = 0.047), as shown in Fig. 2.

### 3.4 Univariate and multivariate analyses of prognostic factors in HCC

To evaluate whether PAR2 expression was an independent risk factor for outcomes in HCC, both univariate and multivariate analyses were conducted. Univariate analysis indicated that TNM stage (*P* = 0.017), vascular invasion (*P* < 0.001) and PAR2 expression (*P* = 0.027) were prognostic factors for OS in patients with HCC. The multivariate analysis showed that vascular invasion (P < 0.001) and PAR2 expression (*P* = 0.032) were independent prognostic factors for OS (Table 2).

**Table 2:** Univariate and multivariate analyses of variables for overall survival

Variables	Univariate analysis				Multivariate analysis		
	HR	95% CI	P		HR	95% CI	P
Age	0.918	0.668–1.262	0.598				
Sex	0.739	0.459–1.191	0.215				
AFP	1.121	0.788–1.594	0.525				
Cirrhosis	1.184	0.804–1.743	0.393				
Tumor size, cm	1.100	0.786–1.538	0.579				
Differentiation	0.824	0.518–1.312	0.415				
TNM stage	1.094	1.002–1.709	0.017				
Vascular invasion	2.642	1.751–3.985	< 0.001		2.118	1.648–3.845	< 0.001
PAR2 expression	1.427	1.062–2.118	0.027		1.347	1.041–1.914	0.032

Abbreviations: *PAR2* proteinase activated receptor 2, *AFP* alpha-fetoprotein

We further explored the risk factors associated with DFS (Table 3) and HCC recurrence (Table 4). Univariate analysis showed that tumor size (*p* = 0.022), vascular invasion (*p* = 0.002) and PAR2 expression (*p* = 0.030) were risk factors associated with DFS. Multivariate analysis also showed that tumor size (*p* = 0.035), vascular invasion (*P* = 0.007) and PAR2 expression (*P* = 0.032) were independent risk factors associated with DFS. For HCC recurrence, the multivariate analysis indicated that vascular invasion (*P* = 0.026) and PAR2 expression (*P* = 0.048) were independent risk factors associated with HCC recurrence.

**Table 3:** Univariate and multivariate analyses for disease-free survival

Variables	Univariate analysis				Multivariate analysis		
	HR	95% CI	P		HR	95% CI	P
Age	0.856	0.554–1.322	0.482				
Sex	0.614	0.314–1.203	0.155				
AFP	1.005	0.633–1.595	0.984				
Cirrhosis	1.278	0.742–2.204	0.377				
Tumor size, cm	1.130	1.066–1.526	0.022		1.012	1.008–1.479	0.035
Differentiation	1.602	0.816–3.144	0.816				
TNM stage	1.022	0.555–1.884	0.555				
Vascular invasion	1.869	1.004–3.512	0.002		1.824	1.004–3.194	0.007
PAR2 expression	1.007	1.003–1.462	0.030		1.002	1.002–1.521	0.032

Abbreviations: *PAR2* proteinase activated receptor 2, *AFP* alpha-fetoprotein

**Table 4:** Univariate and multivariate analyses for recurrence

Variables	Univariate analysis				Multivariate analysis		
	HR	95% CI	P		HR	95% CI	P
Age	0.929	0.586–1.471	0.752				
Sex	0.624	0.307–1.269	0.193				
AFP	0.863	0.536–1.391	0.546				
Cirrhosis	1.182	0.679–2.057	0.555				
Tumor size, cm	0.985	0.583–1.663	0.955				
Differentiation	1.379	0.694–2.737	0.359				
TNM stage	1.096	0.566–2.122	0.786				
Vascular invasion	1.919	1.069–3.696	0.001		1.827	1.051–2.885	0.026
PAR2 expression	1.057	1.005–1.631	0.032		1.048	1.002–1.379	0.048

Abbreviations: *PAR2* proteinase activated receptor 2, *AFP* alpha-fetoprotein

## Discussion

HCC is a common malignant tumor with high mortality in China [28, 29]. Early diagnosis and liver resection may improve the clinical outcome of HBV-related HCC patients. However, due to the high rate of recurrence, the prognosis of HCC patients is still poor.

PARs are proteins coupled to the G protein [22]. The expression of PAR2 is significantly increased in digestive tract tumors and is involved in tumour proliferation, invasion and metastasis [18–21].. In our study, we found that PAR2 showed differential expression between HCC and paired liver tissues. Furthermore, high expression of PAR2 was associated with poorer differentiation and advanced TNM stage, which indicated PAR2 might participate in the progression of HBV-related HCC. In addition, our study indicated that HCC patients with high PAR2 expression had both decreased OS and DFS.

PAR2 is ubiquitously expressed in various tissues of the digestive system [30, 31]. Some studies also indicated that PAR2 is increased in malignant tumors such as breast cancer, lung cancer and esophageal cancer [19, 32–34].

The results of this study confirmed that the expression of PAR2 is increased in HCC tissues making PAR2 a potential prognostic biomarker and therapeutic target in HCC. However, the mechanism by which PAR2 promotes HCC progression remains to be elucidated.

There are some limitations in our study. Since it is a retrospective study, limited data available not allowed to investigate the potential relationship between HBV infection and PAR2. Moreover, immunohistological measurement of PAR2 expression has been performed by a semiquantitative methodic. Further studies on larger cohort of patients will allow to validate the role of PAR2 in HCC and its potential prognosis prediction value. In summary, our data showed that PAR2 expression was increased in HCC. High PAR2 expression was correlated with both decreased OS and DFS in patients with HCC and served as an independent factor for poor prognosis.

## Data Availability

Authors can confirm all relevant data are included in the article and materials are available on request from the authors.
